# Development of a framework with tools to support the selection and implementation of patient-reported outcome measures

**DOI:** 10.1186/s41687-019-0171-9

**Published:** 2019-12-30

**Authors:** Philip J. van der Wees, Eva W. Verkerk, Marjolein E. A. Verbiest, Marloes Zuidgeest, Carla Bakker, Jozé Braspenning, Dolf de Boer, Caroline B. Terwee, Ildikó Vajda, Anna Beurskens, Simone A. van Dulmen

**Affiliations:** 1 Radboud University Medical Center, Radboud Institute for Health Sciences, IQ Healthcare, Nijmegen, the Netherlands; 2 Tilburg University, Tilburg School of Social and Behavioral Sciences, Tilburg, The Netherlands; 3 National Health Care Institute, Diemen, the Netherlands; 4 Leiden University Medical Center, Leiden, the Netherlands; 5 Netherlands Federation of University Medical Centres (NFU), Utrecht, the Netherlands; 6 Netherlands Institute for Health Services Research (Nivel), Utrecht, the Netherlands; 7 Department of Epidemiology and Biostatistics, Amsterdam Public Health Research Institute, Amsterdam UMC, Vrije Universiteit Amsterdam, Amsterdam, the Netherlands; 8 VSOP Dutch Genetic Alliance, Soest, the Netherlands; 9 STZ-Dutch Association of Top Clinical Hospitals, Utrecht, the Netherlands; 10 Maastricht University, Maastricht, the Netherlands; 11 Zuyd University of applied sciences, Heerlen, the Netherlands

**Keywords:** Patient reported outcomes (PROs), Patient reported outcome measures (PROMs), Quality of care, Shared decision-making, Quality improvement, Public reporting, Transparency

## Abstract

**Background:**

Patient reported outcomes (PROs) provide information on a patient’s health status coming directly from the patient. Measuring PROs with patient reported outcome measures (PROMs) has gained wide interest in clinical practice for individual patient care, as well as in quality improvement, and for providing transparency of outcomes to stakeholders through public reporting. However, current knowledge of selecting and implementing PROMs for these purposes is scattered, and not readily available for clinicians and quality managers in healthcare organizations. The objective of this study is to develop a framework with tools to support the systematic selection, implementation and evaluation of PROs and PROMs in individual patient care, for quality improvement and public reporting.

**Methods:**

We developed the framework in a national project in the Netherlands following a user-centered design. The development process of the framework contained five iterative components: (a) identification of existing tools, (b) identification of user requirements and designing steps for selection and implementation of PROs and PROMs, (c) discussing a prototype of the framework during a national workshop, (d) developing a web version, (e) pre-testing of the framework. A total of 40 users with different perspectives (clinicians, patient representatives, quality managers, purchasers, researchers) have been consulted.

**Results:**

The final framework is presented as the PROM-cycle that consists of eight steps in four phases: (1) goal setting, (2) selecting PROs and PROMs, (3) developing and testing of quality indicator(s), (4) implementing and evaluating the PROM(s) and indicator(s). Users emphasized that the first step is the key element in which the why, for whom and setting of the PROM has to be defined. This information is decisive for the following steps. For each step the PROM-cycle provides guidance and tools, with instruments, checklists, methods, handbooks, and standards supporting the process.

**Conclusion:**

We developed a framework to support the selection and implementation of PROs and PROMs. Each step provides guidance and tools to support the process. The PROM-cycle and its tools are publicly available and can be used by clinicians, quality managers, patient representatives and other experts involved in using PROMS. Through periodic evaluation and updates, tools will be added for national and international use of the PROM-cycle.

## Introduction

Patient reported outcomes (PROs) are aspects of a patient’s health status directly reported by the patient. PROs are assessed with patient-reported outcome measures (PROMs), questionnaires consisting of one or multiple questions. PROMs differ from patient reported experience measures (PREMs), which measure experiences of patients with processes of care. Following the increase of PROM use in clinical studies, PROMs have gained wide interest in clinical practice for use in individual patients, as well as for comparing and improving clinician or organizational quality of care through performance measures (PRO-PM) [1–3]. However, available information about how to select, measure and compare PROs and PROMs is scattered, and clinicians or quality managers interested in measuring PROs are having a hard time getting an overview of this process. Many organizations and stakeholders at local, national and international level are seeking support for establishing routine collection of PROM data for use in individual patient care, and for comparing and improving quality of care.

PROMs can play an important role in patient-centered healthcare [1, 4]. Discussing PROM scores in the patient-clinician interaction could improve the communication, patient engagement and self-efficacy as the patient is more involved in goal setting [5–7]. In addition, individual PRO data aggregated across patients can be used as quality indicators to improve quality of care, and for providing transparency of outcomes of care to stakeholders through public reporting of performance measures [1–3]. In summary, PRO measurement is expected to contribute to high value healthcare [8].

Several tools exist that guide parts of the process of PROM selection and use. The COSMIN initiative has developed methodology for selecting the most suitable PROM for measuring a selected PRO, based on the measurement properties [9, 10]. The International Society for Quality of Life (ISOQOL) has recommended minimum standards for the measurement properties of PROMs used in patient-centered outcomes research [11]. These guidelines focus on the measurement properties of PROMs. ISOQOL also developed a user’s guide to implementing PRO assessment in clinical practice [12]. In addition, a users’ guide to integrating patient-reported outcomes in electronic health records has been developed by Johns Hopkins University, with ISOQOL as collaborating partner [13]. However, these guides provide limited support for organizations wanting to implement PRO measurement for patient care, quality improvement or public reporting.

Based on national discussions with PROM users and experts, and a survey among patient representatives of the Netherlands Patients Federation, a need was felt for a framework guiding the process for selecting and implementing PROs and PROMs in different contexts and for different purposes. To meet this unmet need, we wanted to provide an overview with practical guidance through a cyclic and visual framework. Primary target users are clinicians, patient representatives, and quality managers who want to start using PROMs in daily practice or want to evaluate their current use of PROMs. The framework may also be relevant for other stakeholders, including researchers, purchasers and policy makers. Thus, the framework functions as a stepwise, cyclic approach for new users; and as a reference guide for creating awareness in current users and stakeholders.

The aim of this study was to develop a framework to support the selection and implementation of PROs and PROMs in individual patient care, for quality improvement, and for public reporting.

## Methods

### Design and setting

The development of the framework was initiated by the National Health Care Institute and the Netherlands Federation of University Medical Centers. The National Health Care Institute maintains the quality, accessibility and affordability of healthcare in the Netherlands on behalf of the Ministry of Healthcare. We developed the framework in a national project using an iterative, user-centered design, i.e. we consulted end-users throughout the design, development, and testing phase of the framework [14]. We conducted this project in a country-specific approach. We wanted to provide a framework that adheres to international standards for the selection and use of PROMs, and also applicable to the Dutch context. The primary aim was to make the framework available at national level in the Netherlands, while we also aimed at international applicability through adaptation to other international contexts. The framework was developed between January 2016 and March 2017.

### Structure of the development

A project group including 11 experts in the field of PROMs [SvD, EV, MV, MZ, CB, JB, DdB, CT, IV, AB, PvdW] developed the framework through six bimonthly interactive sessions. The project group was purposively established from members of a national network of PROM experts: the PROM-platform. The PROM-platform is a national network of 40 expert members including clinicians, patient representatives, quality managers, purchasers, policy makers and researchers, representing the potential target group of end-users. The expertise of the members of the project group represented diverse perspectives in terms of expertise in selecting and using PROMs [AB, SvD, PvdW], methodological expertise [CT, DdB], healthcare policy [MZ], and perspectives of patients [IV] and clinicians [CB, JB]. Two group members [EV, MV] prepared the sessions and drafted the components and content of the framework. The framework was developed in an iterative process based on the outcomes of the sessions. The PROM-platform monitored the development process and provided feedback on the interim results. Interim results were also fed back to representatives of the professional bodies of medical specialists, hospitals and nurses in healthcare and the Netherlands Patients Federation.

### Patient and public involvement

One of the experts in the project group [IV] represented the parent body of patient organizations in the Netherlands (Netherlands Patients Federation). Six patient representatives (5 female, 1 male) were included in the study through the PROM-platform and in a national workshop, with one patient representative of the Netherlands Patients Federation, four patient representatives of specific patient advocate groups (Parkinson’s disease, kidney disease, cancer, genetic & congenital disorders), and one patient representative from a hospital. Three patient representatives were core participants of the PROM-platform, and the three other representatives were interested participants of the national workshop. All patient representatives except for the hospital representative were employed by their organizations as staff member (*n* = 3) or director (*n* = 2). The patient representatives were experienced in their role as patient advocates through interactions with researchers and policy makers.

### Development process

The development process contained five iterative components in which the project group and the PROM-platform designed and tested the framework. The components are summarized in Table 1.

**Table 1:** Development process of the framework

#	Description
1	Identification of existing tools such as checklists, methods, handbooks and standards for the selection of PROs and PROMs
2	Identification of user requirements and designing a step-by-step approach for the selection and use of PROs and PROMs, providing a framework for end-users
3	Presenting and discussing a prototype of the framework during a national workshop
4	Designing and developing a web version for the framework with guidance and tools included
5	Testing and finalizing the framework in a project in which PROs and PROMs were selected for patients with osteogenesis imperfecta; and through interviews with potential end-users

#### Identifying existing resources

We identified existing national and international tools, such as checklists, methods, handbooks, and standards for the selection and use of PROs and PROMs. We searched the websites of relevant institutes with expertise in PROs and PROMs and asked the project group to deliver materials they were familiar with. We selected national and international tools that contained the most relevant information and were most user-friendly. In selecting the tools we considered their applicability by clinicians, patient representatives, and quality managers as the primary target users, while other stakeholders can use the framework and tools as reference guide.

Clinicians are focusing on patient outcomes that are presented in clinical guidelines or can be supportive to (shared) decision making. From the patient perspective, PROs should be a starting point to optimize daily functioning and dealing with the consequences of the disease. Quality managers are in search for key information to describe the performance of the clinical practice (e.g. in hospitals or nursing homes). Purchasers are in need for information for performance-based contracting, they therefore would like to compare different interventions on their outcomes. Policy makers want to be able to follow patient outcomes over time to see how the outcomes are related to their policy. Researchers like to study appropriate PRO tools from all different perspectives. When each of these user types initiates a project for the selection and use of PROs and PROMs, they should have a thorough understanding of available tools to ensure sufficient expertise for conducting the project. End-users who use PROMs in their daily practice may use the tools to fill their individual knowledge gaps.

#### Designing a step-by-step approach

Based on the tools identified, we designed a framework in a cyclic step-by-step approach for the selection and use of PROs and PROMs. The framework is presented as the PROM-cycle and guides users through the different steps, offering supportive tools for each step. We identified user requirements and established an overview of the process. Steps in the framework that were more complex and require specific methodology were explained in more detail, thus providing specific guidance for each step in the PROM-cycle. End-users may consider consulting experts for conducting more complex steps of the framework, which are specified in each step.

#### Presenting and discussing prototype of the framework

The prototype of the PROM-cycle was presented and discussed during a national workshop, attended by 110 participants. This workshop was initiated in collaboration with ISOQOL Netherlands. Participants were invited to comment on the prototype in different sessions during the workshop.

#### Development of web version of the framework

Based on the outcomes of the workshop, we designed a prototype web version of the PROM-cycle during an interactive session of the project group with a web designer. Consequently, the website was developed and its content was reviewed by two web-content experts.

#### Testing and finalizing the framework

We tested the prototype PROM-cycle in a project in which PROs and PROMs were selected for patients with osteogenesis imperfect (steps 1–4). The results are described in Additional file 1. In addition, interviews were held with seven potential end-users to evaluate the content of the framework. These end-users were four researchers, two quality officers and one clinician. The framework was evaluated on three areas: 1) the content of the PROM-cycle with its four phase and eight steps, 2) the added value of the tools with instruments, checklists, methods, handbooks, and standards provided in the PROM-cycle; 3) the lay-out and usability of the website. The steps of the framework were deemed relevant and feasible in the project for the selection of PROs and PROMs for patients with osteogenesis imperfecta. The interviews with potential end-users resulted in several editorial changes, and examples were added to provide further practical guidance to the different steps in the framework.

## Results

**Fig. 1:**
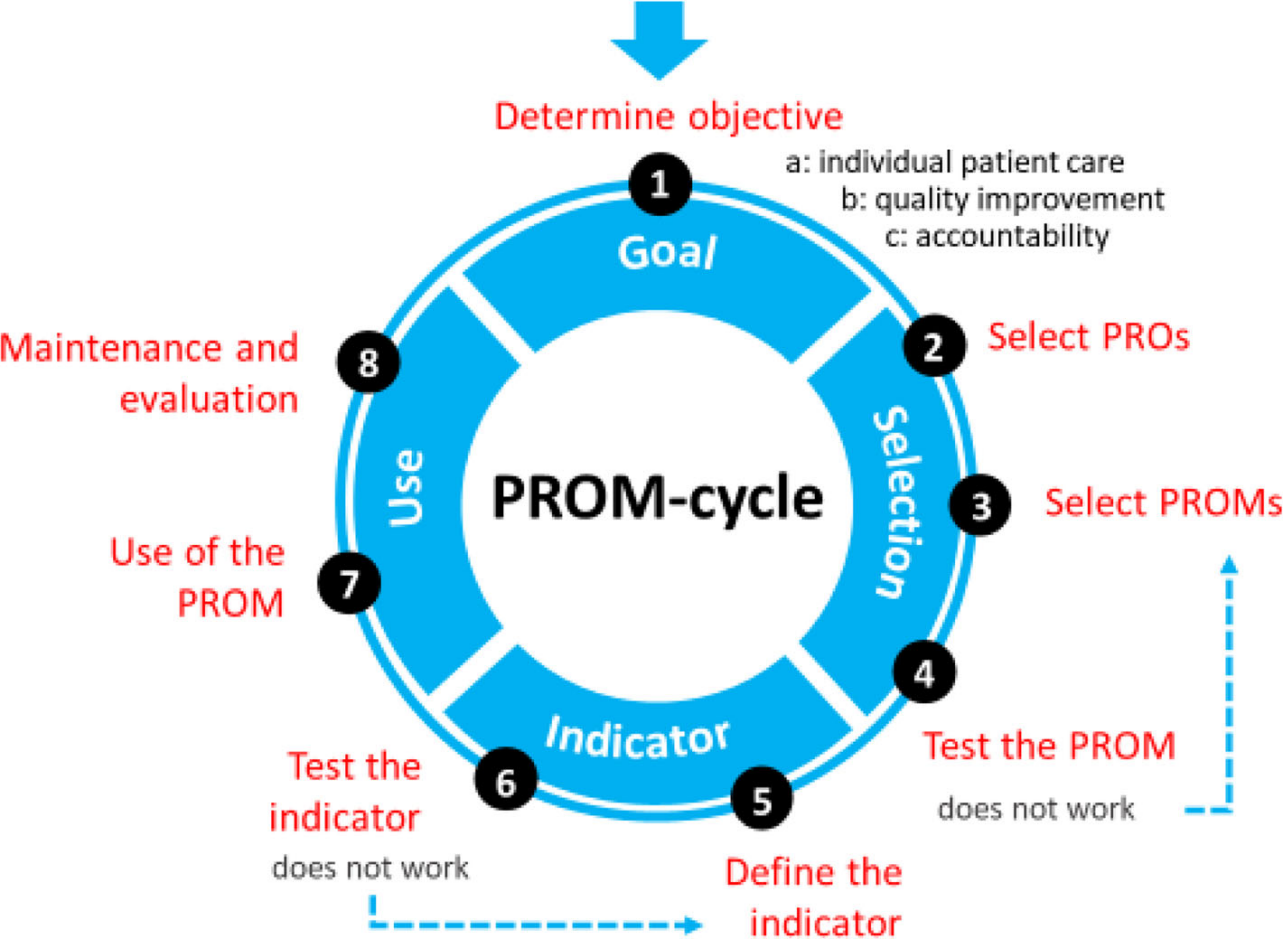
The PROM-cycle

We successfully completed all components of the development process and created a cyclic framework with eight steps divided into four phases; the PROM-cycle. The phases of the PROM-cycle include: (1) goal setting for PRO measurement, (2) selection of PROs and PROMs, (3) developing and testing of quality indicator(s), (4) using and evaluating the PROM(s) and indicator(s). The full PROM-cycle is presented in Fig. 1.

Key tools identified in the development process informed the development of the framework. These documents include the COSMIN methodology for selecting PROMS [9, 10], the ISOQOL standards for using PROMs in patient-centered outcomes research, [11] and ISOQOL’s user’s guide to implementing PRO assessment in clinical practice [12]. We used the pathway of the National Quality Forum for developing PRO based performance measures [15], which includes two background papers [16, 17]. We also used methods and standards published by the American Medical Association for developing PRO based performance measures [18–20]. Specific methodology reports are integrated in the tools of the PROM-cycle.

Setting a clear goal for the use of PROs and PROMs (phase 1) was considered very important for the subsequent steps. Since the project group and PROM-platform experienced that many initiatives for using PROs and PROMs lack a clear objective, we decided to emphasize this phase. Furthermore, an overall orientation of all phases and steps is recommended before starting. Such an orientation may assist in providing an overall picture of the complexity in selecting and implementing PROs and PROMs.

Although the phases and steps are presented sequentially, we emphasize the cyclic approach in the selection and use of PROs and PROMs for their different purposes. Once started with using the steps in the cycle it may be necessary to switch back and forth between the different steps during the process, or even skip steps. Each step is elaborated by a description of its content. The tools provided for end-users in each step include checklists, methods, handbooks, standards, examples of projects, and links to relevant websites. The PROM-cycle is presented through an interactive website which allows for browsing through each of the eight steps with guidance and links to the tools. An English version of the PROM-cycle is also provided, with guidance and tools for each step of the cycle available in a PDF-document.

A more detailed description of the phases and steps within the PROM-cycle is described below.

### Phase 1: determine the objective for using PROs and PROMs

A key component and first step in selecting and using PROs is to determine why, for whom and in which setting the PRO(s) will be used (step 1). The goal, target group and setting will guide all of the following steps. Phase 1 of the PROM-cycle provides a reference guide with tools to support end-users in defining the objectives, target group and setting for using PROs and PROMs.

We distinguish three main objectives for using PROs and PROMs in clinical practice: (A) individual patient care, (B) quality improvement, (C) public reporting [1, 2]. Objectives for using PROs and PROMs may also be combined. Literature, expert opinion and experiences with initiatives for using PROMs all seem to point out that individual patient care is a primary objective, while quality improvement and public reporting may follow. The use of PROMs starts in individual patient care and can then be aggregated at group level for quality improvement and public reporting.

- Objective A: In individual patient care for goal setting, shared decision making, monitoring, and/or evaluation of care. Individual PROM scores can be used in the patient-clinician interaction and for self-management of patients.
- Objective B: For quality improvement using data of groups of patients. PROM scores of groups of patients can be used as management information by care organizations to evaluate outcomes of care, and for quality improvement initiatives.
- Objective C: For providing insight in outcomes of care to stakeholders through public reporting. PROM scores of groups of patients can be made public and used for comparing quality between care organizations. In making performance reports available to the public, patients may use such information in choosing providers, while purchasers may use the outcomes for performance-based contracting.

### Phase 2: selection of PROs and PROMs

In phase 2, which includes steps 2–4 of the PROM-cycle, the user determines which relevant PRO(s) will be measured (step 2) and which PROMs will be used for measuring the selected outcome(s) (step 3). In using the framework it is important to first identify what PROs should be measured, before selecting a PROM to ensure that the selected PROM really measures the topics of interest. However, in practice it may be that PROMs are already routinely collected. The framework can then be used retrospectively to verify whether the PROM actually measures the important PROs, and whether it addresses the goal(s) for which PROs are measured.

The selected PROM should then be tested to determine whether it does match the users’ needs regarding validity and reliability and to assess the patients’ and clinicians’ experiences (step 4). Depending on the goal and context of using the PROM, different aspects of validity and reliability may be important. For example: if a PROM is used as screening instrument it should have good sensitivity and specificity; while responsiveness is important if a PROM is used to evaluate changes over time. The PROM-cycle provides tools for these steps, including conducting a literature search for PROs and PROMs, selecting PROs and PROMs, and consensus methods.

The selection of PROs (step 2) allows for considering self-reported aspects of health to be measured. Health outcomes may include symptoms (e.g. pain), functional status (e.g. physical functioning) or quality of life. Various frameworks are available that classify health outcomes, such as the International Classification of Functioning, Disability and Health [21], the model by Wilson and Cleary [22], and the Patient Reported Outcomes measurement Information System (PROMIS) conceptual framework [23]. Most frameworks distinguish physical, mental and social aspects of health. We did not choose one specific framework for selecting PROs but we present relevant frameworks and provide guidance for their use.

The selection of PROs should be linked to the purpose(s) of measuring the outcomes. The selection process is usually based on a search of the literature followed by a consensus procedure by stakeholders. It may vary from a rigorous systematic literature review and formal consensus procedure to a quick scan of the literature and informal consensus procedure. The rigor may vary based on available budget or existing knowledge and consensus on the outcomes to be selected.

For selecting the PROMs (step 3) we have distinguished a number of sub-steps which are summarized in Table 2. These sub-steps are required due to the complexity of selecting a PROM. These sub-steps include determining the requirements of the PROM, identifying and selecting potential PROMs, evaluating the measurement properties of the PROM, determining feasibility, interpretability and acceptability of the PROM, and finally selecting the PROM and determining the next step. The rigor of the selection of PROMs and the use of the sub-steps may vary, and depends on the goals, specific context and resources of the organization in which the PROMs are used. The selection is based on trade-offs between ideal and actual characteristics of PROMs, and the circumstances in which they are used. Content validity (step 3c) is important to ensure that the items and domains of the PROM reflect the PROs to be measured. This requires input from patients and clinicians. Additional expertise may be required for conducting the sub-steps in the selection of PROMs. This may be related to methodological expertise in searching and reviewing scientific literature (step 3b), methodological expertise for evaluating the content validity and measurement properties of the potential PROMs (steps 3c-e), and experience in conducting consensus procedures (step 3f) [9].

**Table 2:** Sub-steps in selecting the PROM (step 3 of the PROM-cycle)

Sub-step		Description
3a	Determine requirements of the PROM	Requirements may include the type of PROM (e.g. generic of specific), mode of administration (e.g. paper/pencil, web-based), measurement properties (see also 3d), applicability (see also step 3e).
3b	Identify potential PROMs	Identification of PROMs per selected PRO through a systematic search of the literature and existing databases.
3c	Initial selection of potential PROMs	Initial selection through content validity by evaluating the relevance, comprehensiveness, and comprehensibility of the items and domains of the PROM – does the PROM measure the selected PROs?
3d	Determine measurement properties of the PROM	Evaluate the reliability, validity and responsiveness of the potential PROMs. The COSMIN methodology may assist in the evaluation.
3e	Determine feasibility, interpretability and acceptability of the PROM	Feasibility is related to various aspects such as the time of administration (number of items), availability of translated version, and costs for using the PROM; interpretability is related to meaningful use of the scores on the PROM such as minimal clinical important difference; acceptability is related to support of patients and clinicians in using the PROM
3f	Select suitable PROM(s)	Selection of the PROM(s) based on the outcomes of step 3d and 3e, for which formal and informal consensus procedures can be used.
3g	Determine the next step	The next step could be testing of the selected PROM (step 4 in the PROM-cycle), adaptation of an existing PROM through further development and testing; or development of a new PROM if no existing PROMs meet the requirements.

*PRO* Patient Reported Outcome, *PROM* Patient Reported Outcome Measure, *COSMIN* COnsensus-based Standards for the selection of health Measurement Instruments [9]

In step 4, the selected PROMs are tested in practice to evaluate the reliability, validity and feasibility of PROMs for the goal(s), target group and setting. For testing the PROM in the setting in which it is used, context specific data can be used for evaluating measurement properties, and experiences of patients and clinicians can be collected through interviews. Questions that can be asked are: “Is the PROM feasible for use in clinical practice”; “Are the items of the PROM relevant for the specific patient group?”

### Phase 3: developing a quality indicator

In this phase, which includes steps 5 and 6 of the PROM-cycle, aggregate (group level) PROM data, resulting from testing the measure in step 4, are transformed into a quality indicator (step 5). The quality indicator is tested for its reliability and validity in step 6 to allow interpretation of the outcomes in relation to the quality of healthcare. The PROM-cycle provides tools and examples for developing quality indicators. Additional methodological expertise may be required for conducting these steps. Adequate methodology should be used to develop quality indicators that are valid and reliable for evaluating the quality of healthcare [24].

A quality indicator can be used for monitoring healthcare and to consequently assess changes in clinical practice [24]. Preferably, quality indicators are applicable to large groups of patients and are able to show improvement potential [25]. Healthcare organizations can use quality indicators for internal quality improvement purposes (objective B). When used for public reporting (objective C) indicators can be used by different stakeholders. Requirements for reliability and validity of quality indicators for public reporting are high because the data is publicly available and can be used by purchasers or patients to select a care provider [15]. Such requirements are related to comparability of the collection of PRO data and patient population between care organizations, sufficient sample size, and case-mix adjustment.

A quality indicator is sometimes quantified and expressed as a percentage. The denominator usually describes the target group to which the indicator is applicable. In the numerator, the number of ‘correct’ scores is described, resulting in a percentage of correct scores. In Table 3 we have provided an example of a quality indicator for pain management after surgery. The example relates to pain management after surgery, with the norm that > 80% of the patients should have a pain level of ≤4 at 24 h after surgery measured with a numeric rating scale (NRS). If a hospital does not meet the norm it may need to evaluate and improve its pain management policy or educate clinicians in pain management.

**Table 3:** Example of a quality indicator

Definition	The proportion of patients who underwent surgery and have a pain score ≤ 4 at 24 h after surgery
Rationale	Pain reduction is an important goal in care after surgery. Pain management should be aimed at reducing pain to an acceptable level of ≤4 on a numeric rating scale (NRS).
Numerator	The number of patients who underwent surgery and had a pain measurement ≤4 at 24 h after surgery
Denominator	All patients who underwent surgery and had a pain measurement at 24 h after surgery
Specification	Pain is measured in all patients at 24 h after surgery, using an 11-point numeric rating scale (NRS) with 0 points being no pain at all, and 10 points being unbearable pain
Norm	> 80% of the patients should have a pain level of ≤4 at 24 h after surgery

The testing of the quality indicator (step 6) includes evaluating whether it is feasible to compare PRO data between different care organizations or providers and whether the measured differences are clinically relevant.

### Phase 4: using the PROM

In this phase, which includes steps 7 and 8 of the PROM-cycle, the PROM and if applicable the quality indicator, are used for their specific goal(s) (step 7), and periodically evaluated (step 8).

Large scale implementation of the PROM and indicator can be challenging due to many practical, organizational, cultural or other contextual barriers [26]. Some of these barriers may have been identified during step 4 when testing the PROM or during step 6 when testing the quality indicator. The experience during these testing phases can be used to overcome barriers in implementing the PROM and indicator. It may be necessary to address barriers at the level of clinicians and patients (e.g. knowledge, skills or attitudes about PRO measurement), at the organizational level (e.g. integrating the PRO measurement in the workflow, availability of software for PRO measurement), or at the health system level (regulations such as privacy of data use).

An important facilitator for the implementation of PROMs is to have support of all stakeholders (including but not limited to patients, clinicians, managers, payers) for the use of the PROM. Another important facilitator is providing timely and relevant feedback of individual and group PROM scores to the end- users, such as patients and clinicians. Providing meaningful feedback is essential in making PRO measurement successful [27–29].

## Discussion

We developed the PROM-cycle, providing a framework with step-by-step guidance and useful tools for selecting and implementing PROs and PROMs. The eight steps of the framework, divided in four phases, can be used in a cyclic approach and brings together knowledge and tools currently available for PRO measurements in individual patient care, for quality improvement, and for public reporting. Our user-centered design, stakeholder involvement, and pilot testing provide a basis for further implementation of the PROM-cycle. The framework is currently tested on a larger scale with two projects for the development and implementation of indicators for patient with low-back pain and Chronic Obstructive Pulmonary Disease (COPD) in primary physical therapy care.

The PROM-cycle provides a practical approach with visual representation of the cyclic steps. Its application should be informed by current evidence for the use of PROs and PROMs for their different purposes. Greenhalgh and colleagues have published a realist review identifying underlying mechanisms and contextual factors for the use of PROMs [27]. The review shows that PROMs can have a positive impact on the quality of care. However, it is difficult to draw robust conclusions about how and when PROMs are effective in individual patient care or for improving healthcare quality. Users of the PROM-cycle should be aware that the context is very important in formulating hypotheses and objectives of using PROs and PROMs. An important gap in the literature is the lack of knowledge about using PROMs for quality improvement [28]. The body of knowledge in this field is in its early phases of development, and we hope that the PROM-cycle will support in building knowledge in this field.

Using PROMs in individual patients for screening, goal setting, monitoring and/or evaluation is an important prerequisite for routine data collection, ensuring that patients and clinicians will put effort in collecting reliable and valid data. The data of individual patients can then be aggregated at group level for quality improvement or public reporting. In addition, the feedback of group level data is considered more useful when such data is also used in individual patients. As a result, clinicians are more inclined to use feedback reports for quality improvement initiatives. An integrated approach addressing the different objectives may thus enhance the implementation of PRO measurement [1, 27].

### Strengths and limitations

The main strength of our study is that we developed the PROM-cycle in close partnership with the various end-users, including clinicians, quality managers, patient representatives, and researchers. This user-centered approach enabled end-users to provide feedback throughout the development process of the framework. This feedback strengthened our awareness for the need of the framework and is likely to empower end-users in the uptake of the PROM-cycle.

This study has several limitations. One limitation is that we only tested the framework in limited projects and further testing of the value of the PROM-cycle is needed. Another limitation of our study is that the PROM-cycle has been developed for use in the Netherlands. However, we used international reference documents and guidance and we translated the PROM-cycle and guidance into English, thus making the framework available for international validation. Several tools provided in the framework are derived from international publications. We expect that the steps in the framework are likely to be generic for international use, while country-specific tools and examples provided may require adaptations. The need for guidance in selecting and using PROs and PROMs is also recognized internationally, and has resulted in tools of the National Quality Forum (NQF) [15] and the Physician Consortium for Performance Improvement (PCPI) [19]. Our framework also refers to these documents and we think our hands-on approach may also be of added value for international use.

The framework is limited to measuring and comparing health outcomes through PROMs, and does not include the use of patient experiences with processes of care as measured with PREMs. We think the cyclic approach and the steps in the framework may also be applicable for the selection and use of PREMs, but was not the focus of our project. PROMs and PREMs are related in the sense that they reflect the perspectives of patients, and they can both be used for measuring quality of care. However, they represent different constructs of healthcare quality, and they also differ in their use in clinical practice. The potential application for using the framework in selecting and using PREMs should therefore be tested.

### Implications for research, policy and practice

The PROM-cycle is publicly available. This may make selecting and using PROMs more accessible for clinicians and quality managers in healthcare organizations that are new to the field of PROMs. The framework also emphasizes the need for sufficient expertise when initiating the use of PROMs in clinical practice. In addition, since the framework provides information about common pitfalls and how to prevent them, it may help PROM initiatives to become more successful.

The next steps are to further disseminate, evaluate and improve the PROM-cycle. The dissemination of the PROM-cycle includes presentations at conferences and workshops with end users. The international validity of the framework can be further tested by presenting and discussing the framework in international meetings. Additional tools and country-specific examples may be considered at country level. Evaluation and adaptation of the PROM-cycle is scheduled annually, thus making the framework dynamic. Through periodic updates, tools will be added for national and international use of the PROM-cycle.

## Conclusion

The PROM-cycle is a user-friendly and practical framework that supports the selection and implementation of PROs and PROMs in clinical practice. Each step provides guidance and tools to support the process. The PROM-cycle and its tools are publicly available and can be used by clinicians, quality managers and other professionals involved in using PROMS. The next steps are to disseminate, evaluate and update the PROM-cycle periodically based on large scale testing.

### Website

Dutch version:

https://www.zorginzicht.nl/kennisbank/Paginas/prom-toolbox.aspx

English version:

https://www.zorginzicht.nl/kennisbank/Paginas/prom-toolbox-english-summary.aspx

## Supplementary information

**Additional file 1.** Presents the results of testing the framework for steps 1-4 of the PROM-cycle. The framework was tested for selecting PROs and PROMs for patients with Osteogenisis Imperfecta to be used in a web-based program for PROMs data collection. KliK is a web-based program using electronic PROMs with the aim to monitor and screen children (aged 0–18) with chronic illnesses over extended periods of time. For patients with Osteogenisis Imperfecta there are no PROMs included yet. The aim of the project was to identify PROMs for this patient group.

## Data Availability

All data generated or analyzed during this study are included in this published article, its supplementary information files, and the publicly available website of the study.

## Abbreviations

ISOQOL: International Society for Quality of Life
PRO: Patient Reported Outcome
PROM: Patient Reported Outcome Measure
