# Supplementary material for: Development of a framework with tools to support the selection and implementation of patient-reported outcome measures
Source: J Patient Rep Outcomes. 2019 Dec 30;3:75. doi: 10.1186/s41687-019-0171-9 (PMC6937349; doi:10.1186/s41687-019-0171-9)
Supplement: Supplementary file 1 — Additional file 1. Presents the results of testing the framework for steps 1-4 of the PROM-cycle. The framework was tested for selecting PROs and PROMs for patients with Osteogenisis Imperfecta to be used in a web-based program for PROMs data collection. KliK is a web-based program using electronic PROMs with the aim to monitor and screen children (aged 0–18) with chronic illnesses over extended periods of time. For patients with Osteogenisis Imperfecta there are no PROMs included yet. The aim of the project was to identify PROMs for this patient group. [file 41687_2019_171_MOESM1_ESM.docx]

**Additional file 1. Results of testing the framework (steps 1-4 of the PROM-cycle)**

**Background**

KliK is a web-based program using electronic PROMs with the aim to monitor and screen children (aged 0–18) with chronic illnesses over extended periods of time. The questionnaires in KLIK are available prior to a consultation. Pediatricians retrieve the ePROfile from the website (www.hetklikt.nu) and discuss it with the patients. The ePROfile consists of a literal representation of the answers and a graphic presentation. Various tools are used to aid in its interpretation. All members of the multidisciplinary team receive training in how to use the website and how to adequately respond to the patient’s ePROfile. For patients with Osteogenesis Imperfecta (IO) there are no PROMs included yet. The aim of the project was to identify PROMs for this patient group. The testing of the framework resulted in four recommended potential combinations of selected PROMs (see table 3). It shows that the selection of PROMs is based on trade-offs between ideal and actual characteristics of PROMs and circumstances in which they are used.

**Phase 1**

Step 1: determining objective

Result: For use in individual patient care

**Phase 2**

Step 2: selection of PRO

Methods:

1. Literature review: systematic reviews on quality of life in patients with OI and search in guidelines of OI
2. Two focusgroup with patients and clinicians

Result: Relevant PROs

1. not being able to do everything by yourself
2. pain
3. self confidence
4. social contacts

Other important PRO’s:

1. independence
2. functioning at school

**Phase 3**

Step 3: selection PROMs

Results:

Table 1 with properties of identified PROMs

Table 2 with PROs included in the selected PROMs

Table 3 with four recommended potential combinations of selected PROMs

**Table 1: Properties of identified PROMs**

|  | | **HUI-II** | **SPPC** | **Mont-petit** | **PEDI** | **Wee-FIM** | **CIS-20** | **PODCI** | **Peds-QoL** | **CHQ** | **Pain** | **Peers** | **Friend-ship** | **Stigma** |
| --- | --- | --- | --- | --- | --- | --- | --- | --- | --- | --- | --- | --- | --- | --- |
| **General characteristics** | **Nr of items** | 17 | 36 | 20 | 197 | <197 | 20 | 87 | 23 | 87 | 2 | 8 | 5 | 8 |
|  | **Number of domains** | 5 |  | 5 | 3 | 2 | 4 | 4 | 4 | 11 | 2 | 1 | 1 | 1 |
|  | **Dutch version** | yes | yes | nee | yes | yes | yes | yes | yes | yes | yes | no | no | no |
|  | **Already in KLIK** | yes |  |  |  |  |  |  | yes |  | yes |  |  |  |
|  | **Validated group** | Cancer | healthy | not | Physical disability | Physical disability | CFS | Physical disability | psychiatry | JAI |  | patients | patients | patients |
| **Clinimetric characteristics** | **Reproducibility** |  | + |  | + | + |  | + | + | +/- |  |  |  |  |
|  | **Internal consistency** |  | + |  | + |  | + |  | + (6-7 +/-) | + |  |  | + | + |
|  | **Content validity** |  | + |  | + | + |  |  |  | + |  | + |  |  |
|  | **Convergent validity** | + | + |  |  |  |  | + | + |  |  |  |  | + |
|  | **Responsivity** |  |  |  |  |  |  |  |  | + |  |  |  |  |
|  | **Interpretability** |  |  |  |  |  | + |  |  |  |  |  |  |  |
|  | **Precision** |  |  |  |  | + | + | + |  |  |  |  |  |  |
|  | **Acceptability** |  |  |  |  |  |  |  |  |  |  |  |  |  |
|  | **Feasibility** | +/- | +/- | - | - | - | +/- | +/- | + | +/- | + |  |  |  |

**Table 2: PROs included in the selected PROMs**

|  | **HUI-II** | **SPPC** | **Montpetit** | **PEDI** | **WeeFIM** | **CIS-20** | **PODCI** | **PedsQL** | **CHQ** | **Pijn** | **Peers** | **Friendship** | **Stigma** |
| --- | --- | --- | --- | --- | --- | --- | --- | --- | --- | --- | --- | --- | --- |
| **Social contacts** |  | + |  | + | + |  | + |  | + |  | + | + |  |
| **School** |  | +/- |  |  |  |  |  | + |  |  |  |  |  |
| **Pain** |  |  |  |  |  |  | + | +/- | + | + |  |  |  |
| **Independence** |  |  | + |  |  |  |  |  |  |  |  |  |  |
| **Not being able to do everything yourself** | + |  | + | ++ | ++ |  | + | + | + |  |  |  |  |
| **Self confidence** |  | + |  |  |  |  | + |  | + |  |  |  |  |
| **Acceptation** |  |  |  |  |  |  |  | + |  |  |  |  |  |
| **Variety** |  |  |  |  |  |  |  |  |  |  |  |  |  |
| **Fear** |  |  |  |  |  |  |  |  |  |  |  |  |  |
| **Incomprehension** |  | + |  |  |  |  |  | + |  |  |  |  | + |
| **Access** |  |  | + |  |  |  |  |  |  |  |  |  |  |
| **Fatigue** |  |  |  |  |  | + |  | +/- |  |  |  |  |  |

| **Table 3: Properties of four recommended potential combinations of PROMs** |
| --- |

|  | | **PedsQL**  **PODCI**  **Pain** | **PedsQL**  **CHQ**  **Pain** | **PedsQL**  **Peers**  **Pain** | **PedsQ LFriendship**  **Pain** |
| --- | --- | --- | --- | --- | --- |
| **General** | **Items** | 43 | 40 | 39 | 36 |
|  | **remarks** | Not validated | Not validated | Not available in Dutch | Not available in Dutch |
| **PROs** | **Social contacts** | X | X | X | X |
|  | **School** | X | X | X | X |
|  | **Pain** | X | X | X | X |
|  | **Independence** |  |  |  |  |
|  | **Not being able to do everything yourself** | X | X | X | X |
|  | **Self confidence** | X | X |  |  |
|  | **Acceptation** | X | X | X | X |
|  | **Variety** |  |  |  |  |
|  | **Fear** |  |  |  |  |
|  | **Incomprehension** | X | X | X | X |
|  | **Access** |  |  |  |  |
|  | **Fatigue** |  |  |  |  |
